# Accelerated age‐related cortical thinning in mild traumatic brain injury

**DOI:** 10.1002/brb3.1161

**Published:** 2018-11-28

**Authors:** Priya Santhanam, Steffanie H. Wilson, Terrence R. Oakes, Lindell K. Weaver

**Affiliations:** ^1^ Lovelace Biomedical Research Albuquerque New Mexico; ^2^ The Emmes Corporation Rockville Maryland; ^3^ Madison School of Medicine and Public Health University of Wisconsin Madison Wisconsin; ^4^ Division of Hyperbaric Medicine Intermountain Medical Center Murray, UT and Intermountain LDS Hospital Salt Lake City Utah; ^5^ Department of Medicine University of Utah School of Medicine Salt Lake Utah

**Keywords:** aging, cortical thinning, mild traumatic brain injury

## Abstract

**Introduction:**

Mild traumatic brain injury (mTBI) can result in many structural abnormalities in the cerebral cortex. While thinning of the cortex has been shown in mTBI patients, there is high regional variability in reported findings. High‐resolution imaging can elucidate otherwise unnoticed changes in cortical measures following injury. This study examined age‐related patterns of cortical thickness in U.S. active duty service members and veterans with a history of mTBI (*n* = 66) as compared to a normative population (*n* = 67).

**Methods:**

Using a fully automated cortical parcellation methodology, cortical thickness measures were extracted from 31 bilateral cortical regions for all participants.

**Results:**

The effect of diagnosis and age on cortical thickness (group × age interaction) was found to be significant (*p* < 0.05) for many regions, including bilateral parietal and left frontal and temporal cortices. Findings held for a male‐only subset, and there was no effect of time since injury in any regions.

**Conclusions:**

The presence of mTBI appeared to accelerate age‐related cortical thinning across the cortex in our study population.

## 1 INTRODUCTION

The prevalence of mild traumatic brain injury (mTBI) in the active duty and veteran populations is significant (DVBIC, 2015; Terrio et al., 2009; Warden, 2006). A survey from 2012 of returning veterans previously stationed in Iraq or Afghanistan found 17% reported an mTBI during deployment, with 59% of those reporting multiple injuries (Wilk, Herrell, Wynn, Riviere, & Hoge, 2012). MTBI has been defined as physical trauma causing disruption to brain function, resulting in a brief change in mental status (disorientation, confusion, memory loss, or loss of consciousness for <30 min) and including observable signs of neurological dysfunction (ACRM, 1993). Service members who suffer at least one mTBI event often have persistent neurocognitive and neurological issues (Bryant & Harvey, 1999; Carroll et al., 2004; Schneiderman, Braver, & Kang, 2008), which are generally known as postconcussive symptoms (PCS). These include depression, anxiety, insomnia, headache, dizziness, and tinnitus (Bryant & Harvey, 1999). A recent study showed increased rates of dementia related to TBI, specifically a diagnosis occurring 1.5 years earlier on average in the mTBI population as compared to those without injury history (Barnes et al., 2018). Unlike with moderate or severe TBI, mTBI patients appear radiologically normal clinically and, on average, suffer less severe cognitive deficits and less progressive atrophy over time (Affairs., 2009; Mac Donald et al., 2011; Tate & Bigler, 2000). However, a multitude of recent high‐resolution imaging studies of mTBI report significant and persistent neuroanatomical alterations, including atrophy, diffuse axonal injury, and neuronal degeneration (Bigler & Maxwell, 2012; Inglese et al., 2005; MacKenzie et al., 2002). In particular, the emergence of high‐field magnetic resonance imaging (MRI) has aided in a more complete picture of cortical and cytoarchitectural changes following injury (Arciniegas, Anderson, Topkoff, & McAllister, 2005; Bigler & Maxwell, 2012).

Brain volumetric changes are well‐characterized in those with a history of mTBI. Global and regional atrophy have been reported following mTBI (Bigler, 2015; Bigler & Maxwell, 2012; Mayer, Hanlon, & Ling, 2015), even many years postinjury. While gray and white matter volumetric changes are often reported with mTBI (Bigler, 2013; Tate, Khedraki, Neeley, Ryser, & Bigler, 2011), there have not been many studies examining changes specific to the cortical surface. Thickness of the cerebral cortex, a lesser utilized metric of neuroanatomy, reflects underlying regional gray matter integrity and is hypothesized to be geometrically related to both cortical surface area and cortical volume (Van Essen, Drury, Joshi, & Miller, 1998; Winkler et al., 2010). Changes or abnormalities in cortical thickness have been reported in populations with drug abuse disorders, neurological disease, and brain injury (Hutton, Vita, Ashburner, Deichmann, & Turner, 2008). Some studies of TBI have reported abnormalities in cortical thickness after injury (Govindarajan et al., 2016; King, Lopez‐Larson, & Yurgelun‐Todd, 2016; Michael et al., 2015; Tate et al., 2014), but findings were regionally variable and inconsistent across acute and chronic mTBI populations.

Changes to the cerebral cortex can also occur in normal aging. Many regions experience cortical thinning as part of the normal aging process (Fjell et al., 2009; Salat et al., 2004). However, given the known effects of injury on cortical measures, it is possible for mTBI to exacerbate the normal cortical thinning that is present in older age. To this end, the present study examined the effects of age and mTBI on cortical thickness in regions comprising the entire cortex. We hypothesized that older individuals with a history of mTBI would have greater thinning of the cortex than older individuals with no mTBI history.

## 2 METHODS

### 2.1 Participants

Participants were active duty U.S. service members and veterans recruited as part of a prospective study of postconcussive symptoms following mTBI (Weaver et al., 2018; Weaver, Chhoeu, Lindblad, Churchill, & Wilson, 2016). Inclusion criteria for the mTBI group required a history of at least one mild traumatic brain injury (mTBI) with persistent symptoms that met all the following criteria: brain injury that occurred more than 3 months prior to baseline screening at the local site, with the most recent injury occurring no more than 5 years prior to randomization; most recent TBI occurred on active duty; TBI was caused by nonpenetrating trauma or blast exposure; TBI resulted in at least one of the following at the time of injury: a period of loss of or a decreased level of consciousness (up to 30 min), a loss of memory for events immediately before or after the injury (up to 24 hr), or alteration in mental state at the time of the injury (becoming dazed or confused); and has current complaints of symptoms such as headache, dizziness, or cognitive or affective problems.

Head injury eligibility was determined by the Ohio State University TBI Identification Method (Bogner & Corrigan, 2009; Corrigan & Bogner, 2007), a structured interview administered by site coordinators used to obtain the number and nature of self‐reported lifetime TBIs as well as the frequency and severity of postconcussive symptoms. The study was conducted at three local study sites: Joint Base Lewis‐McChord, Washington; Fort Carson, Colorado; and Camp Lejeune, North Carolina. Participants were recruited from these sites and evaluated for TBI eligibility using the OSU; baseline neuroimaging was conducted for all participants at a common Outcomes Assessment Center at Fort Carson. A normative control group without any history of mTBI was recruited at the Fort Carson, Colorado, site for comparison. A subset of the normative group with ages similar to the mTBI population (maximum age = 53 years) was used for group comparison (*n* = 6 excluded to age match). Excluded from the final analysis were six normative participants in order to age match, as well as five mTBI and two normative participants for poor image quality. Table 1 indicates the final participant group demographic (age and gender) and injury characteristics (time since injury and injury and blast count) for those with (*n* = 66) and without mTBI (*n* = 67). Also included in this table is the self‐reported duration of loss of consciousness for the mTBI group as related to the qualifying injury.

**Table 1:** Demographic characteristics for participants

	Age (years)	Gender	Time since injury (months)	Injury count	Blast count	Duration of loss of consciousness (percentage of total *n*)		
						None	<5 min	5–30 min
mTBI (*n* = 66)	33.2 ± 7.3 (21–53)	65 M, 1 F	23.6 ± 16 (4–60)	3.36 ± 2.3 (1–12)	1.72 ± 1.6 (0–7)	34/66 (51.5%)	26/66 (39.4%)	6/66 (9.1%)
Normative (*n* = 67)	34.8 ± 10.3 (18–53)	47 M, 20 F						

Note

Age, time since injury, injury count, and blast count are displayed as mean  standard deviation (range).

### 2.2 Image acquisition and processing

T1‐weighted anatomical images were acquired on a 3T Philips Achieva MRI system using an ultrafast spoiled gradient echo sequence. The following acquisition parameters were used for the four echo train sequence: TR/TE1/delta TE = 9.3/1.65/1.8 ms along with a 1 × 1 × 1‐mm resolution. Preprocessing steps included the reconstruction of DICOM images (2D images to 3D volumes), masking 3D volume data, resampling, and conversion to Freesurfer input format.

Cortical reconstruction and volumetric segmentation was performed with the Freesurfer image analysis suite (version 5.3). The technical details of these procedures are described in prior publications; briefly, this processing includes removal of nonbrain tissue, (Segonne et al., 2004), automated Talairach transformation, segmentation of the subcortical white matter and deep gray matter volumetric structures (Fischl et al., 2002; Fischl, Salat, et al., 2004) intensity normalization (Sled, Zijdenbos, & Evans, 1998), tessellation of the gray matter–white matter boundary, automated topology correction (Fischl, Liu, & Dale, 2001; Segonne, Pacheco, & Fischl, 2007), and surface deformation following intensity gradients along the gray/white and gray/cerebrospinal fluid borders (Dale & Sereno, 1993; Dale, Fischl, & Sereno, 1999; Fischl & Dale, 2000). Once the cortical models are complete, among other procedures performed was parcellation of the cerebral cortex into regional units (Desikan et al., 2006; Fischl, Kouwe, et al., 2004). This method produces representations of cortical thickness, calculated as the closest distance from the gray/white boundary to the gray/CSF boundary at each vertex on the tessellated surface (Fischl & Dale, 2000). For data analyzed in this study, all surfaces were visually checked thoroughly to ensure that the automated reconstruction was successful, and manual interventions were used as needed to correct small defects. If image defects were considered too extensive, the dataset was deemed poor quality and excluded from the study analysis.

### 2.3 Post hoc statistical analysis

Cortical thickness in millimeters was compared between mTBI and normative groups in 31 bilateral cortical regions. The effect of diagnosis and age on cortical thickness was examined using a univariate linear model for each selected region of the left and right cerebral cortex with cortical thickness measurement as the dependent variable and age at study enrollment and study group (mTBI vs. normative) as independent factors. Main effects of study group and age, as well as interaction of study group and age, were examined in the model. Separate models also examined the potential confounding effect of time since injury (in months) on the association of age and cortical thickness in the mTBI group. Post hoc statistical analyses were performed using SPSS (v.26). This exploratory analysis was intended to be hypothesis‐generating, and as such, no adjustments were made for multiple testing.

## 3 RESULTS

**Figure 1:**
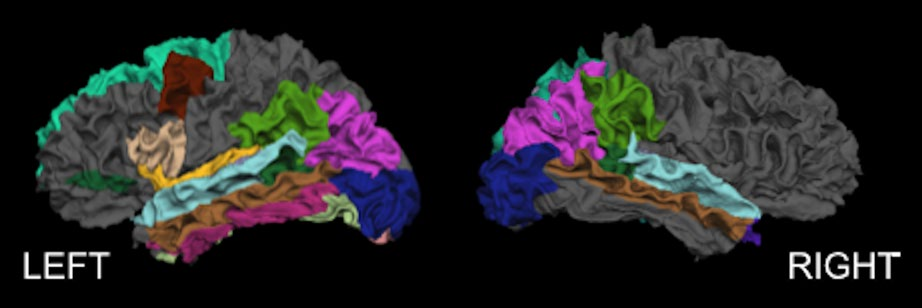
Cortical surface rendering highlighting regions with significantly increased age‐related cortical thinning with mTBI

**Figure 2:**
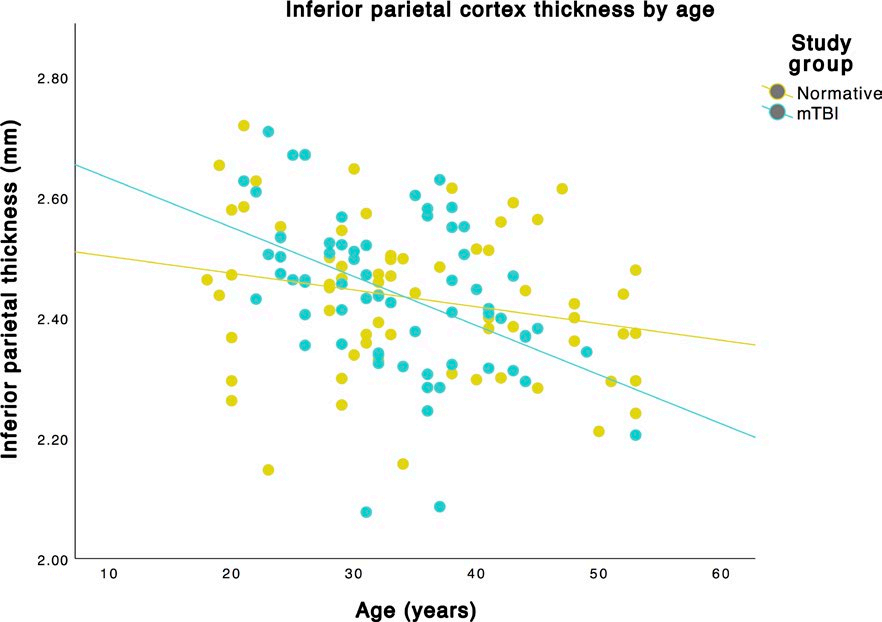
Plot of left hemisphere inferior parietal cortex thickness by age. The steeper slope of the mTBI group indicates a greater thinning with age in this group

Many regions on the left and right cerebral cortex had a greater cortical thinning for older adults with mTBI compared to normative controls (significant interaction of age and study group; Figure 1 and Table 2). Of these, the parietal regions appeared to show the pattern most bilaterally, while many other subregions had a greater effect of cortical thinning with age in those with mTBI compared to normative controls specifically on the left hemisphere. A separate analysis including only males from each group produced mostly the same results (Table 3); the only differences were the right pars orbitalis, paracentral, and pericalcarine regions showed an interaction effect, while left pars opercularis no longer did. Using the left inferior parietal region as an example, we have included a plot of cortical thickness by age (Figure 2) clearly showing a greater decrease in cortical thickness across age for those with mTBI (as compared to the normative population). The magnitude and direction of the differential age effects between the mTBI and normative groups were similar for other regions. Time since injury had no effect on the model of cortical thickness and age in the mTBI group (results not shown), and there were no regions with a significant increase in cortical thickness (i.e., cortical thickening) with age (results not shown).

**Table 2:** Effects of age and study group (interaction) on cortical thickness by cortical region

Cortical region	Left		Right	
	F	p	F	p
Frontal				
Caudal middle frontal	8.355	**0.005**	3.45	0.066
Lateral orbitofrontal	2.946	0.088	0.203	0.653
Medial orbitofrontal	0.991	0.321	0.027	0.871
Paracentral	1.394	0.24	4.384	**0.038**
Pars opercularis	4.413	**0.038**	0.09	0.765
Pars orbitalis	13.272	**0.0004**	3.88	0.051
Pars triangularis	3.347	0.07	2.336	0.129
Precentral	2.9	0.091	3.154	0.078
Rostral middle frontal	1.487	0.225	0.483	0.488
Superior frontal	7.065	**0.009**	3.85	0.052
Frontal pole	0.542	0.463	0.001	0.978
Caudal anterior cingulate	0.235	0.629	0.885	0.349
Rostral anterior cingulate	0.316	0.575	0.421	0.517
Parietal				
Inferior parietal	4.788	**0.03**	10.325	**0.002**
Postcentral	1.15	0.286	0.653	0.421
Precuneus	5.151	**0.025**	6.18	**0.014**
Superior parietal	3.69	0.057	5.816	**0.017**
Supramarginal	5.212	**0.024**	7.612	**0.007**
Isthmus cingulate	0.647	0.423	1.929	0.167
Posterior cingulate	3.077	0.082	4.529	**0.035**
Temporal				
Banks of superior temporal sulcus	6.929	**0.01**	12.124	**0.001**
Entorhinal	0.014	0.906	0.606	0.438
Fusiform	5.192	**0.024**	2.803	0.097
Inferior temporal	5.63	**0.019**	2.279	0.134
Middle temporal	6.129	**0.015**	7.148	**0.008**
Parahippocampal	2.707	0.102	0.755	0.387
Superior temporal	13.118	**0.0004**	15.048	**0.0002**
Temporal pole	0.601	0.44	0.13	0.719
Transverse temporal	0.821	0.367	0.86	0.355
Occipital				
Cuneus	4.603	**0.034**	10.706	**0.001**
Lateral occipital	6.755	**0.01**	6.874	**0.01**
Lingual	2.469	0.119	0.36	0.549
Pericalcarine	2.351	0.128	4.64	**0.033**

Note

Significant interaction of age and study group is shown in bold.

**Table 3:** Effects of age and study group (interaction) on cortical thickness in male‐only subset population

Cortical region	Left		Right	
	F	p	F	p
Frontal				
Caudal middle frontal	7.192	**0.008**	2.871	0.093
Lateral orbitofrontal	2.688	0.104	0.129	0.720
Medial orbitofrontal	0.414	0.521	0.21	0.648
Paracentral	1.767	0.187	4.624	**0.034**
Pars opercularis	3.035	0.084	0.008	0.930
Pars orbitalis	11.67	**0.001**	4.707	**0.032**
Pars triangularis	1.944	0.166	2.248	0.137
Precentral	3.198	0.076	2.972	0.088
Rostral middle frontal	0.932	0.336	1.062	0.305
Superior frontal	7.718	**0.006**	4.705	**0.032**
Frontal pole	0.311	0.578	0.249	0.619
Caudal anterior cingulate	0.001	0.973	0.308	0.580
Rostral anterior cingulate	0.368	0.545	0.236	0.628
Parietal				
Inferior parietal	5.427	**0.022**	9.794	**0.002**
Postcentral	1.302	0.256	1.043	0.309
Precuneus	5.457	**0.021**	5.672	**0.019**
Superior parietal	4.082	0.046	5.614	**0.020**
Supramarginal	5.602	**0.020**	7.628	**0.007**
Isthmus cingulate	1.501	0.223	3.362	0.069
Posterior cingulate	2.894	0.092	4.539	**0.035**
Temporal				
Banks of superior temporal sulcus	5.851	**0.017**	10.34	**0.002**
Entorhinal	0.054	0.817	0.594	0.443
Fusiform	6.581	**0.012**	3.308	0.072
Inferior temporal	4.865	**0.029**	1.856	0.176
Middle temporal	6.911	**0.010**	8.397	**0.005**
Parahippocampal	3.218	0.076	1.857	0.176
Superior temporal	10.84	**0.001**	13.93	**0.0003**
Temporal pole	0.770	0.382	0.535	0.466
Transverse temporal	1.333	0.251	0.781	0.379
Occipital				
Cuneus	4.167	**0.044**	9.383	**0.003**
Lateral occipital	4.867	**0.029**	7.472	**0.007**
Lingual	3.258	0.074	0.689	0.408
Pericalcarine	0.99	0.322	3.764	0.055

Note

Significant interaction of age and study group is shown in bold.

## 4 DISCUSSION

The presence of mTBI appeared to increase age‐related cortical thinning in several regions across the cortex in our study population. Generally, more posterior/rostral regions of the brain showed the accelerated thinning pattern, as compared to anterior/frontal regions. Effects were somewhat left hemisphere lateralized overall, with the parietal lobe having the most bilateral regions affected.

Some previous studies of mTBI have demonstrated time‐dependent differences in cortical recovery (Cole, Leech, Sharp, & Neuroimaging, 2015; Ewing‐Cobbs et al., 2016; Rowe et al., 2016), including both structural and functional reorganization. However, in our study population, time since injury was not a significant factor in the effect of age and mTBI on cortical thickness in any of the cortical regions. Furthermore, given evidence of a gender difference in brain anatomy patterns with aging (Coffey et al., 1998), we examined the age by group interaction in a subset of males only. The majority of findings were consistent in these subgroups, with only a few regional differences mostly in the right hemisphere. Taken together, the findings in the full study population and subset of males support a consistent and robust effect of mTBI on cortical thickness with age.

Previous studies of mTBI have shown variable cortical thinning patterns following injury. A small study of blast‐related mTBI showed thinning of the left superior temporal and frontal gyri (Tate et al., 2014), while another study that included mild and moderate TBI patients showed thinning in primarily the right insula and inferior temporal and frontal regions (Michael et al., 2015). In a longitudinal study of mTBI, Govindarajan et al (2016) reported a cortical thinning in the left middle temporal and right superior parietal regions acutely, with left middle temporal thinning persisting at three months postinjury; these regions are included in our findings as well. As this study reports many more significant regions affected by age and mTBI than previous studies of only mTBI, it is possible the effect of aging and injury together accelerates the cortical thinning process. Furthermore, lateralization of cortical thinning has not been widely reported following injury, but in our study population could also be related to the accelerated process or alternatively to localization and extent of injury.

In this study, accelerated cortical thinning was primarily found in temporal and occipital regions bilaterally. Consistent age‐dependent cortical thinning has been reported in prefrontal, frontal, and primary motor regions with relative sparing in the parahippocampal and medial orbitofrontal regions (Fjell et al., 2009; Salat et al., 2004). In contrast, temporal lobe thinning has been inconsistently linked to aging (Fjell et al., 2009; Salat et al., 2004). With the exception of temporal regions, our findings do not generally overlap with normal age‐related patterns of cortical thinning, supporting a combined influence of age and mTBI on the cortical changes seen in our population.

Accelerated age‐related changes in cortical measures have been reported in a number of study populations (Ewing‐Cobbs et al., 2016; Rowe et al., 2016). Two studies of apolipoprotein E epsilon 4 carriers (Espeseth et al., 2008) and individuals with attention‐deficit/hyperactivity disorder (Shaw et al., 2006), respectively, both showed accelerated cortical thinning with age. Additionally, Cole et al (2015) reported accelerated atrophy in patients with mild‐to‐severe TBI, with more effects in those farther from injury date. In contrast, our study did not show an effect of time since injury, but included only mTBI patients, which may show a different non time‐dependent pattern of accelerated injury.

Areas with the most apparent cortical thinning in this study, left temporal and right parietal lobes, are responsible for a number of high‐level cognitive processes. Though it is possible the patients with more cortical thinning would have more cognitive deficits, such a relationship was outside of the scope of this study. Another limitation was the lack of an elderly population to examine the effects of accelerated age‐related cortical thinning in an older population. Heterogeneity of injury in the mTBI population also limited the linking of localized injury to regional cortical thinning patterns. Furthermore, the cross‐sectional nature of the study did not allow for following changes in cortical thickness at the participant level over time. Finally, statistical analyses were performed across many cortical regions but were not adjusted for multiple testing, so the possibility of Type I error cannot be ruled out. However, that the magnitude and direction of the effect of age and study group on cortical thickness were consistent across outcomes strengthens the evidence of our findings.

To our knowledge, this study was the first of its kind to examine age‐related patterns of cortical thickness in U.S. active duty service members and veterans with a known history of mTBI. Robust and widespread findings across some regions of the cortex, even when controlling for gender and time since injury, suggest the possibility of an increased age‐related cortical thinning process which may be characteristic of mTBI. In conclusion, cortical thickness has the potential to serve as a biomarker for accelerated aging effects in patients contending with the long‐term outcomes of mTBI.
